# Impact of polyethylene glycol 400/propylene glycol/hydroxypropyl-guar and 0.1% sodium hyaluronate on postoperative discomfort following cataract extraction surgery: a comparative study

**DOI:** 10.1186/s40662-017-0079-5

**Published:** 2017-05-10

**Authors:** Georgios Labiris, Panagiota Ntonti, Haris Sideroudi, Vassilios Kozobolis

**Affiliations:** 1 Department of Ophthalmology, University Hospital of Alexandroupolis, 68100 Dragana, Alexandroupolis, Greece; 2 Eye Institute of Thrace, Alexandroupolis, Greece

**Keywords:** Cataract, Postoperative regimen, Artificial tears, Surface discomfort index, Systane, Hylocomod

## Abstract

**Background:**

Universal postoperative guidelines for cataract extraction surgery are yet to be introduced. Artificial tears are gaining popularity as an additional integral component of the postoperative regime. The primary objective of this study was to explore the impact of two prevalent artificial tear preparations on postoperative discomfort following cataract extraction surgery.

**Methods:**

A total of 180 patients that underwent cataract extraction surgery were randomly divided into three groups according to their postoperative regime: a) Study group 1 (SG1) received a fixed combination of tobramycin and dexamethasone (FCTD) quid for 3 weeks and, additionally polyethylene glycol 400/propylene glycol/hydroxypropyl-guar quid, for 6 weeks, b) Study group 2 (SG2) received FCTD quid for 3 weeks and, additionally 0.1% sodium hyaluronate provided in the COMOD® device quid, for 6 weeks, and, c) Control Group (CG) received only FCTD quid for 3 weeks. The following indexes were evaluated at three postoperative checkpoints: 1) Subjective discomfort index (SDI) derived from four direct 10-scale Likert-type questions that were addressed to the patient and pertained to: a) foreign body sensation (FBS), b) blinking discomfort (BD), c) stinging sensation (SS), d) tearing sensation (TS), 2) Tear break-up time (TBUT), 3) Central corneal thickness (CCT) and, 4) Central Corneal Sensitivity (CCS).

**Results:**

Both groups showed increased CCT values at the first examination point and reduced CCS values at all examination points. Furthermore, both SGs had better TBUT times at all examination points compared to CG (CG: 8.86 ± 1.08, SG1: 9.59 ± 1.45, CG2: 9.45 ± 1.33, *p* < 0.05). BD was significantly better in both SGs only at the 1^st^ week of examination, while SDI values were better until the 3^rd^ week and only borderline better at 6^th^ week. Lastly, no significant differences were detected between SGs, regarding all parameters, at all examination points.

**Conclusion:**

Polyethylene glycol 400/propylene glycol/hydroxypropyl-guar and 0.1% sodium hyaluronate provided in the COMOD® device seem to be equally efficient in alleviating OSD symptoms following cataract extraction surgery and any of them should be routinely added to the postoperative regime.

**Trial registration:**

ClinicalTrials.gov Identifier: https://clinicaltrials.gov/ct2/show/NCT02558218NCT02558218

**Electronic supplementary material:**

The online version of this article (doi:10.1186/s40662-017-0079-5) contains supplementary material, which is available to authorized users.

## Background

Cataract is a condition that affects a large number of the middle aged population, being responsible for 33% of visual impairment worldwide and nearly 51% of blindness [1]. The treatment of choice is phacofragmentation, either with ultrasound, liquefaction or laser-assisted; and implantation of an artificial monofocal or multifocal lens inside the capsular bag [2–5]. Cataract extraction surgery is a minimally invasive technique that, in the majority of the cases, is done as an outpatient’s service. Moreover, it is considered to have a short and uneventful recovery period. However, published literature revealed only a few studies concerning postoperative irritation symptoms and pain among cataract patients. Furthermore, the results varied, referring to either decreased or increased percentages of patients experiencing adverse effects after phacoemulsification [6, 7].

In fact, the postoperative regime of patients that underwent cataract extraction aims primarily in preventing endophthalmitis, persistent corneal edema and cystoid macular edema [8, 9]. However, apart from these complications, a series of non-vision-threatening adverse events have been identified that cause great discomfort and frustration to the patient, like pain, foreign body sensation, and itchiness. These symptoms are considered to be associated with postoperative inflammation, corneal nerve transection and overall instability of the corneal tear film [10, 11].

Although universal postoperative guidelines for cataract extraction surgery are yet to be introduced, common practices can easily be identified. Most cataract surgeons prefer fixed combinations of antibiotic with corticosteroids for at least three weeks following cataract extraction. In hard nuclei, when intense energy is delivered in the eye, non-steroidal anti-inflammatory drugs (NSAIDs) can be administered as well. Unfortunately, both aforementioned fixed combinations and NSAIDs do not prevent the temporary symptoms of ocular surface disease (OSD) that almost all cataract patients experience. Therefore, artificial tears are gaining popularity as an additional integral component of the postoperative regime in order to alleviate OSD-related symptomatology.

Among the popular artificial tears medications are the: a) polyethylene glycol 400/propylene glycol/hydroxypropyl-guar (Systane Ultra, Alcon, Greece) consisting of Polyethylene Glycol 400 0.4% and Propylene Glycol 0.3% and b) 0.1% sodium hyaluronate provided in the continuous monodose system COMOD® (Hylocomod, Farmex, Greece). COMOD device is an integral airless application system, which enables delivery of multiple sterile doses of a liquid medicinal product. Both medications are considered to be highly effective in dry-eye-disease (DED) and prospectively in moderate and severe OSD. However, both medications have not been comparatively evaluated for their potential beneficial impact on cataract patients.

Within this context, the present study objective was to explore the impact of two contemporary artificial tear preparations on postoperative discomfort following cataract extraction surgery.

## Methods

### Setting

This was a prospective, clinic-based, randomized trial. Study protocol adhered to the tenets of the Helsinki Declaration and written informed consent was obtained from all participants. The Institutional Review Board of the Democritus University of Thrace approved the protocol and the study was conducted at the University Hospital of Alexandroupolis (UHA), Greece, between September and December 2015. Official registration number of the study is: NCT02558218

### Participants

Participants were recruited from the Cataract Service of the UHA in a consecutive-if-eligible basis. Eligibility criteria included diagnosis of senile cataract. Exclusion criteria for all study groups included: diagnosis or evidence of dry-eye-disease (DED), IOP-lowering medications, former incisional surgery, former diagnosis of corneal disease, diabetes, autoimmune or mental diseases. By means of a custom computer randomization program, all participants were randomly assigned to three study groups according to the postoperative regime that was prescribed: a) Study group 1 (SG1) received a fixed combination of tobramycin and dexamethasone (FCTD), (Tobradex, Alcon, Greece) quid for 3 weeks and, additionally Systane Ultra, Alcon, Greece quid, for 6 weeks, b) Study group 2 (SG2) received Tobradex quid for 3 weeks and, additionally Hylocomod quid, for 6 weeks, and, c) Control Group (CG) received only Tobradex quid for 3 weeks.

### Surgical technique

All operations were performed by the same surgeon (G.L.) in a consistent way using the Alcon Infiniti VisionSystem platform (80% continuous amplitude with 350mmHg vacuum limit and 40ml/min aspiration flow rate), as described previously [5]. In brief, by means of a 2.2mm, superior-temporal or superior-nasal (eleven o’clock), self-sealing, clear-cornea incision, 3% Sodium Hyaluronate and 4% Chondroitin Sulfate (Viscoat, Alcon, Greece) were injected for the phacofragmentation phase and 1% Sodium Hyaluronate (Provisc, Alcon, Greece) for the rest of the intraocular lens implantation phase [12]. For all participants, the foldable hydrophilic acrylic intraocular lens SN60WF (Alcon) was inserted in the capsular bag.

### Data collection

The following parameters were comparatively evaluated 1, 3 and 6 weeks postoperatively: 1) Subjective discomfort index (SDI) was derived using four direct 10-scale Likert-type questions (Additional file 1) that were addressed to the patient and pertained to: a) foreign body sensation (FBS), b) blinking discomfort (BD), c) stinging sensation (SS), d) tearing sensation (TS), 2) Tear break-up time (TBUT), 3) Central corneal thickness (CCT) using anterior segment optical coherence tomography and, 4) Central Corneal Sensitivity (CCS) using the Cochet-Bonnet aesthesiometer.

### Validation of the subjective discomfort index

Validation of the SDI was performed in a sample of 40 participants who visited our cornea outpatient service for dry eye disease (DED). These participants populated four validation groups according to their DED severity, as suggested by the Dysfunctional Tear Study group [13, 14]. Construct validity was assessed with one-way analysis of variance (ANOVA) in order to confirm that all indexes could efficiently discriminate validation groups based on their DED progress. All indexes presented significant discriminant ability (*p* < 0.05). Moreover, test-retest reliability was assessed for the four validation groups by calculation of intraclass correlation coefficients (ICCs) for all indexes at two different visits with an average time-window of 1 month, to prevent memory effect. All ICCs were above 0.90 indicating excellent reliability of the indexes.

### Statistical analysis

An *a priori* power analysis was performed. For an effect size of 0.74, 52 participants would be required in each group, for the study to have a power of 0.8 at the significance level of 0.05. The normality of measured data was evaluated by Kolmogorov-Smirnov test. Normal distribution data were assessed by Student’s t-test. Non-parametric data were assessed with Mann–Whitney U test. Multiple comparisons among the three groups were assessed by analysis of variance (ANOVA). Values at the *p* < 0.05 were considered statistically significant. All statistical analyses were performed with the Medcalc version 9.6.2.0 (Medcalc Software, Mariakerke, Belgium).

## Results

One hundred eighty patients (98 men and 82 women, mean age 65.2 ± 11.5 years) were recruited and were randomly assigned SG1 (59 participants), SG2 (60 participants) or CG (61 participants). Detailed demographic and clinical parameters are presented in Table 1. Non-significant differences were detected with respect to age (*p* = 0.33) and BSCVA (*p* = 0.23) among the groups.

**Table 1:** Preoperative data for all participants

Study group	No.	Age		BSCVA	
		Years	SD	LogMAR	SD
*SG1*	59	64.2	11.2	0.65	0.08
*SG2*	60	66.1	10.5	0.60	0.07
*CG*	61	64.8	9.9	0.63	0.11
*p*		*0.33*		*0.23*	

*SG1* = Study group 1; *SG2* = Study group 2; *CG* = Control group; *BSCVA* = Best spectacles corrected visual acuity

**Fig. 1:**
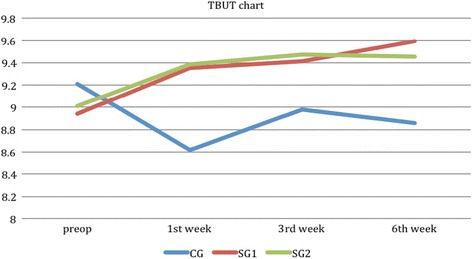
Tear break-up time chart

**Fig. 2:**
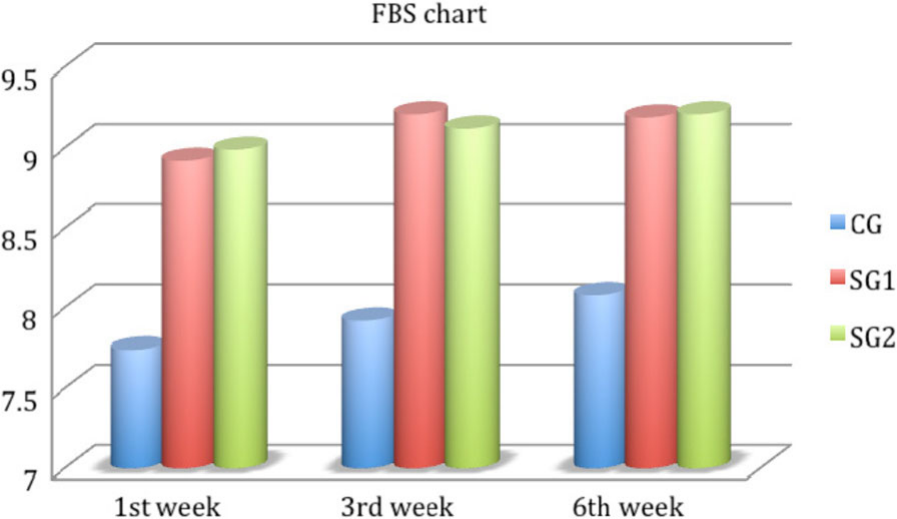
Foreign Body Sensation chart

**Fig. 3:**
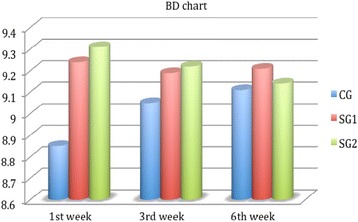
Blinking discomfort chart

**Fig. 4:**
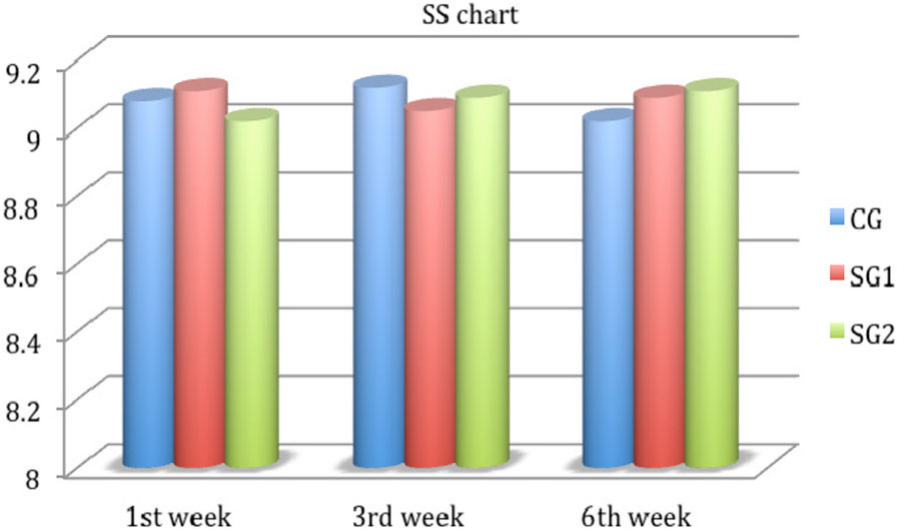
Stinging sensation chart

**Fig. 5:**
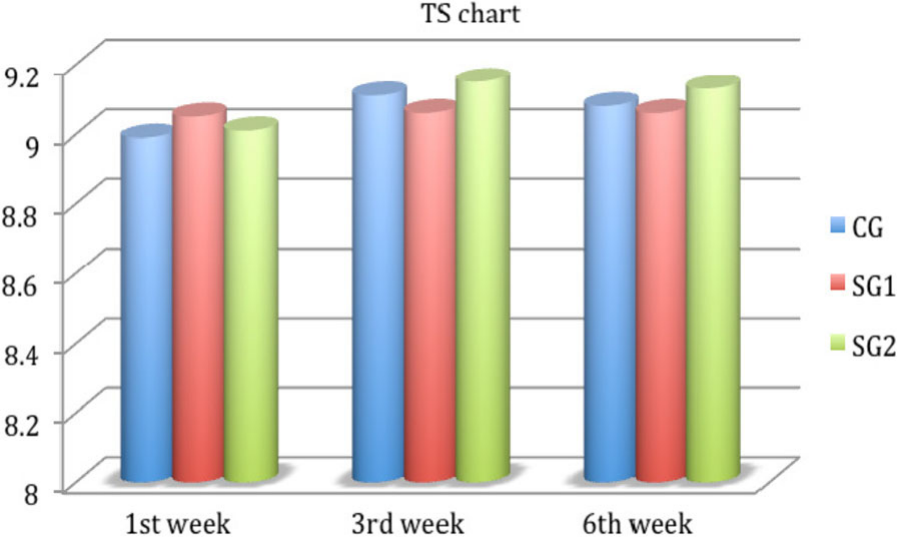
Tearing sensation chart

**Fig. 6:**
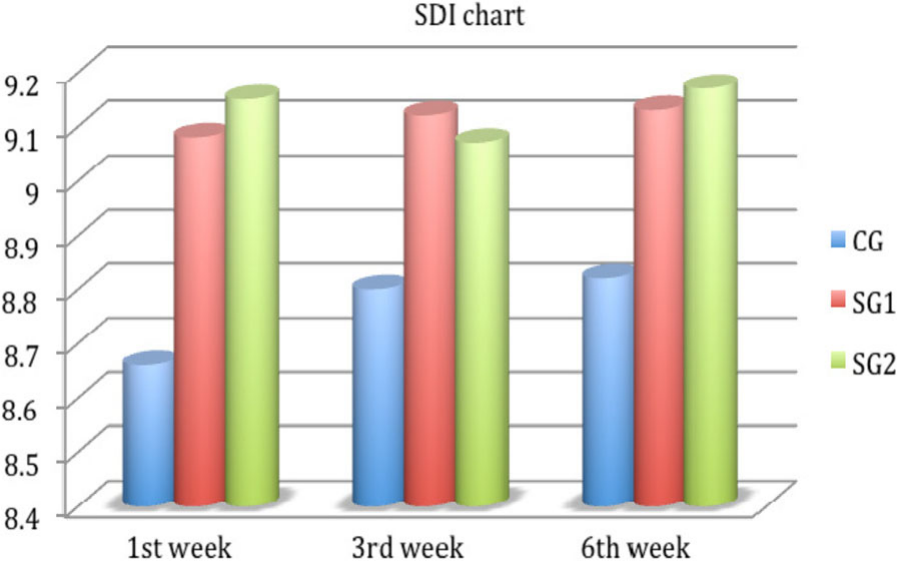
Subjective discomfort index chart

All postoperative comparisons are presented in Tables 2, 3, 4 and 5. Both groups demonstrated significantly increased CCT values at the first examination point and significant reduced CCS values at all examination points. Non-significant correlations were detected between CCT, CCS and SDI components. Regarding TBUT (Fig. 1), study groups demonstrated significantly better times at all examination points in comparison to the control group and to the group’s preoperative value (all *p* < 0.05). TBUT time demonstrated significant correlation with FBS (*r* ^2^ = 0.58, *p* < 0.01), which was significantly better in the study groups for all examination points (Fig. 2). On the other hand, BD was significantly better in both study groups only at the first week (9.24 ± 0.56, 8.85 ± 1.98, *p* = 0.04, Fig. 3), and non-significant differences could be detected for the rest of SDI components at all examination points (Figs. 4 and 5). Accordingly, SG1 and SG2 participants demonstrated significantly better SDI values at the first two postoperative examination visits (until the third week), and borderline better SDI at the last examination visit i.e., 6^th^ week, see Fig. 6. Regarding comparisons between study groups, non-significant differences could be detected for all parameters at all examination visits.

**Table 2:** Group comparisons preoperatively

	Preoperative			
Parameter	CG	SG1	SG2	p
CCT (μm)	529 ± 21	532 ± 28	539 ± 24	0.34
CCS (cm)	5.45 ± 0.72	5.39 ± 0.58	5.41 ± 0.73	0.44
TBUT (secs)	9.21 ± 0.99	8.94 ± 1.11	9.01 ± 1.05	0.35
FBS	NA	NA	NA	NA
BD	NA	NA	NA	NA
SS	NA	NA	NA	NA
TS	NA	NA	NA	NA
SDI	NA	NA	NA	NA

*CCT* = Central Corneal Thickness; *CCS* = Central Corneal Sensitivity; *TBUT* = Tear Break-up Time; *FBS* = Foreign Body Sensation; *BD* = Blinking Discomfort; *SS* = Stinging Sensation; *TS* = Tearing Sensation; *SDI* = Subjective Discomfort Index

**Table 3:** Group comparisons on the 1^st^ week

	1st week			
Parameter	CG	SG1	SG2	p
CCT (μm)	550 ± 36♭	560 ± 28♭	564 ± 41♭	0.16
CCS (cm)	4.41 ± 0.92♭	4.31 ± 1.89♭	4.24 ± 1.55♭	0.11
TBUT (secs)	8.62 ± 1.45♭	9.35 ± 1.34♭	9.38 ± 1.11♭	0.03
FBS	7.74 ± 1.41	8.92 ± 1.39	8.99 ± 1.45	0.02
BD	8.85 ± 1.98	9.24 ± 0.56	9.31 ± 0.62	0.04
SS	9.08 ± 1.67	9.11 ± 1.21	9.02 ± 0.87	0.34
TS	8.99 ± 1.22	9.05 ± 0.88	9.01 ± 0.91	0.23
SDI	8.66 ± 1.57	9.08 ± 1.01	9.15 ± 1.16	0.04

*CCT* = Central Corneal Thickness; *CCS* = Central Corneal Sensitivity; *TBUT* = Tear Break-up Time; *FBS* = Foreign Body Sensation; *BD* = Blinking Discomfort; *SS* = Stinging Sensation; *TS* = Tearing Sensation; *SDI* = Subjective Discomfort Index

♭indicates significant difference with preoperative values

**Table 4:** Group comparisons on the 3^rd^ week

	3rd week			
	CG	SG1	SG2	p
CCT (μm)	541 ± 32	549 ± 38	555 ± 52	0.42
CCS (cm)	4.75 ± 1.14♭	4.69 ± 1.26♭	4.72 ± 1.41♭	0.21
TBUT (secs)	8.98 ± 1.52♭	9.41 ± 1.28♭	9.47 ± 1.13♭	0.01
FBS	7.92 ± 2.15	9.21 ± 1.89	9.12 ± 1.73	<0.01
BD	9.05 ± 1.43	9.19 ± 0.74	9.22 ± 0.97	0.12
SS	9.12 ± 1.47	9.05 ± 0.91	9.09 ± 1.04	0.27
TS	9.11 ± 0.78	9.06 ± 0.77	9.15 ± 0.94	0.34
SDI	8.80 ± 1.46	9.12 ± 1.76	9.07 ± 1.52	0.04

*CCT* = Central Corneal Thickness; *CCS* = Central Corneal Sensitivity; *TBUT* = Tear Break-up Time; *FBS* = Foreign Body Sensation; *BD* = Blinking Discomfort; *SS* = Stinging Sensation; *TS* = Tearing Sensation; *SDI* = Subjective Discomfort Index

♭indicates significant difference with preoperative values

**Table 5:** Group comparisons on the 6^th^ week

	6th week			
	CG	SG1	SG2	p
CCT (μm)	548 ± 29	550 ± 25	543 ± 31	0.36
CCS (cm)	4.82 ± 0.87♭	4.77 ± 1.31♭	4.79 ± 1.26♭	0.26
TBUT (secs)	8.86 ± 1.08♭	9.59 ± 1.45♭	9.45 ± 1.33♭	0.01
FBS	8.08 ± 1.23	9.19 ± 1.65	9.21 ± 1.42	0.01
BD	9.11 ± 0.97	9.21 ± 0.36	9.14 ± 0.47	0.19
SS	9.02 ± 1.15	9.09 ± 0.64	9.11 ± 0.73	0.22
TS	9.08 ± 0.85	9.06 ± 0.59	9.13 ± 0.87	0.28
SDI	8.82 ± 1.05	9.13 ± 0.81	9.17 ± 0.58	0.05

*CCT* = Central Corneal Thickness; *CCS* = Central Corneal Sensitivity; *TBUT* = Tear Break-up Time; *FBS* = Foreign Body Sensation; *BD* = Blinking Discomfort; *SS* = Stinging Sensation; *TS* = Tearing Sensation; *SDI* = Subjective Discomfort Index

♭indicates significant difference with preoperative values

## Discussion

Cataract extraction is considered the most prevalent ophthalmological operation both in the developing and the developed societies [15, 16]. Despite the advances in cataract-extraction techniques and cataract-related technology, a series of intraoperative and postoperative adverse-effects and complications have been associated with this prevalent surgical modality. Among the mild ones are the transient corneal edema and the reduced corneal sensitivity. More severe ones include the permanent corneal decompensation due to endothelial cell damage. However, in the majority of published trials, cataract extraction surgery seems to provide excellent visual outcomes [17] with high levels of patient satisfaction [18]. In fact, the postoperative visual rehabilitation results in an average 2.8 quality-adjusted-life-years (QUALYs) for bilateral cases. Despite this impressive outcome, it is common knowledge that cataract patients do experience dry-eye symptoms of variable severity and variable duration in the postoperative period [19]. Within this context, the present study attempted to explore the beneficial impact of two prevalent artificial tears preparations in preventing or effectively managing dry-eye symptoms.

In order to explore the potential efficacy of each, we reviewed the literature to identify the most common DED symptoms that cataract patients experience following extraction surgery. The most common symptoms from the literature [20–23] were: foreign body sensation, blinking discomfort, stinging sensation, and tearing sensation. These four common DED-related disturbances allowed us to construct an overall discomfort index (the Surface Discomfort Index) that quantified the overall perceived discomfort that the patients experienced. Prior to addressing the questionnaires to the patients, we attempted a validation pre-study, which suggested excellent construct validity and reliability of the index.

Both artificial tears used, Systane Ultra [24, 25] and Hylocomod [26] , are considered highly effective in ocular surface disorders. Systane Ultra, which combines polyethylene glycol 400, propylene glycol and hydroxypropyl-guar, acts through a unique biphasic mechanism of action, in which the product first binds to damaged hydrophobic areas of epithelial cells to add volume to the tear film and then restructures the tear film by forming a protective gel matrix that provides long-lasting protection [27]. Hylocomod contains 0.1% sodium hyaluronate, which mimics the rheological properties of the aqueous layer, hence produces a beneficial effect to the ocular surface by stabilizing it [28].

Our study outcomes indicated significant improvement of the TBUT index for both study groups. TBUT improvement was associated with significant improvement of the SDI index; the latter finding was primarily attributed to the significant reduction of the foreign body sensation for the whole postoperative period and the significant reduction of the blinking discomfort for the first postoperative week. On the other hand, both study groups demonstrated better results, albeit not significant, for the stinging sensation and the tearing sensation when compared to the control group that received only the standard postoperative regime. Moreover, none of the study groups presented significant superiority over the other, for all postoperative examination points. It seems that the biphasic protective mechanism of Systane Ultra provides no additional beneficial impact over the monophasic Hylocomod, at least for the early postoperative period.

Our results are in accordance to former published studies that reported significant improvement of TBUT and foreign body sensation when 0.1% sodium hyaluronate and 0.5% carboxymethylcellulose ophthalmic solution was additionally prescribed for the postoperative regime [29]. In fact, the effective postoperative management of the ocular surface that the additional artificial tears medication provides, improves patient satisfaction significantly with the surgical outcome and fosters the bond between the physician and the patient [30, 31].

## Conclusion

Taking into account the limitations of this study i.e., the short duration of the postoperative assessment and its single-centered design, our statistically robust number of patients suggests that both Systane ultra and Hylocomod are equally efficient in alleviating OSD symptoms following cataract extraction surgery and should be routinely added to the postoperative regime.

## Additional files

Additional file 1: Post-Cataract subjective discomfort index questionnaire (Cat-SDI-Q) ver.1.1. (PDF 75.2 kb)
