# Supplementary material for: Impact of polyethylene glycol 400/propylene glycol/hydroxypropyl-guar and 0.1% sodium hyaluronate on postoperative discomfort following cataract extraction surgery: a comparative study
Source: Eye Vis (Lond). 2017 May 10;4:13. doi: 10.1186/s40662-017-0079-5 (PMC5424421; doi:10.1186/s40662-017-0079-5)
Supplement: Supplementary file 1 — Post-Cataract subjective discomfort index questionnaire (Cat-SDI-Q) ver.1.1. (PDF 75.2 kb) [file 40662_2017_79_MOESM1_ESM.pdf]

Post-Cataract Subjective Discomfort Index Questionnaire (Cat-SDI-Q) ver.1.1

1. **Please evaluate the foreign body sensation in your eye. Use the scale from 1 (severe) to 10 (none).**

1 2 3 4 5 6 7 8 9 10

similar to a sensation of a hot ember in the eye	*similar to the sensation of sand or dust in the eye*	*similar to sensation of fine dust or powder in the eye*

2. **Please evaluate the tearing discomfort of the eye. Use a scale from 1 (severe) to 10 (none).**

1 2 3 4 5 6 7 8 9 10

constant or nearly constant spilling of tears over the lid margin.	*infrequent or intermittent spilling of tears over the lid margin.*	*positive sensation of fullness of the conjunctival sac without tears spilling over the lid margin*

3. **Please evaluate potential pain or discomfort that you experience when blinking your eyes. Use scale from 1 (severe) to 10 (none)**

1 2 3 4 5 6 7 8 9 10

Very intense discomfort or pain. I want to keep my eyes closed most of the time	*I feel pain or discomfort that interferes with my visual capacity and makes me frustrated*	*I feel very rarely a vague discomfort that does not interfere with my visual capacity*

4. **Please evaluate potential stinging sensation that you experience. Use scale from 1 (severe) to 10 (none)**

1 2 3 4 5 6 7 8 9 10

Very intense stinging sensation. I want to keep my eyes closed most of the time	*I have that stinging sensation several times per day. It makes me frustrated*	*I have very rarely a stinging sensation. It doesn't make me worry*

© Georgios Labiris MD
Medical School, Democritus University
